# Global trends and forecasts of cervical cancer and a real-world safety assessment of human papillomavirus vaccines in women: A systematic analysis of the Global Burden of Disease study 2021 and the Vaccine Adverse Event Reporting System database

**DOI:** 10.1371/journal.pone.0345286

**Published:** 2026-03-23

**Authors:** Kai Yan, Guang Yang, Lixuan Yan, Li Wu, Yang Wang, Peifeng He, Qi Yu

**Affiliations:** 1 School of Management, Shanxi Medical University, Taiyuan, China; 2 Shanxi Key Laboratory of AI-Enhanced Clinical Decision Support, Taiyuan, China; 3 Department of Information Technology, Digital Health Guidance Center of Shanxi Province, Taiyuan, China; 4 Department of Pediatrics, Shanxi Medical University, Taiyuan, China; 5 Department of Anesthesiology, Second Hospital of Shanxi Medical University, Taiyuan, China; 6 Department of Anesthesiology, Shanxi Provincial People’s Hospital (Fifth Hospital) of Shanxi Medical University, Taiyuan, China; 7 Xinzhou Vocational and Technical College, Xinzhou, China.; Universidad Autonoma de Yucatan, MEXICO

## Abstract

**Background:**

Cervical cancer (CCA) remains a major cause of morbidity and mortality among women globally, particularly in low- and middle-income countries. Although human papillomavirus (HPV) vaccination is central to prevention, safety concerns may affect vaccine acceptance. We integrated global disease-burden trends with real-world post-marketing safety data to provide complementary public health evidence for CCA prevention.

**Methods:**

Using Global Burden of Disease (GBD) 2021 data, we assessed global prevalence, incidence, death, and disability-adjusted life years (DALYs) for women with CCA from 1990–2021, and applied age–period–cohort (APC) models to characterize temporal patterns. Bayesian APC models were used to project future incidence and death, with retrospective validation. For vaccine safety, adverse event (AE) reports following HPV vaccination in females (Cervarix, Gardasil, Gardasil 9; 2006–2025) were extracted from the Vaccine Adverse Event Reporting System (VAERS). We performed disproportionality analyses using four algorithms to identify reporting signals, with designated medical event (DME) screening and subgroup analyses by age and vaccine type.

**Results:**

Globally, the age-standardized incidence rate of CCA decreased from 18.1 to 15.3 cases per 100,000 women from 1990 to 2021, while new cases increased from 0.41 million to 0.67 million. The highest incidence and death rates were observed in sub-Saharan Africa and selected Pacific Island countries. Projections suggest continued declines in age-standardized incidence and death through 2050, although the absolute burden will likely remain substantial. In VAERS, 41,731 HPV vaccine-related reports were identified; most were non-serious (80.9%). Syncope (ROR = 5.81, 95%CI:5.64–5.99), loss of consciousness (ROR = 5.26, 95%CI: 5.06–5.47) and pallor (ROR = 6.39, 95%CI: 6.10–6.70) were the most frequently reported events, and six potential DME-related signals were detected.

**Conclusions:**

Despite declining age-standardized rates, CCA continues to impose a substantial global burden with marked regional disparities. Sustained HPV vaccine prevention efforts should be supported by epidemiological evidence and transparent, evidence-based safety communication.

## Introduction

Cervical cancer (CCA) is the fourth most common gynecological malignancy globally, accounting for 6.8% of new cancers in women [1]. In addition, CCA is the second leading cause of death in women worldwide [2]. In 2022, 661,000 new CCA cases and 348,200 CCA-related deaths were reported worldwide [3], with 150,700 new cases and 55,700 deaths recorded in China [4]. In 2020, the World Health Organization (WHO) introduced a worldwide plan aimed at “accelerating the elimination of cervical cancer as a public health problem” [5], marking the first time the world has committed to eradicating this cancer type. However, further significant efforts are still needed to resolve this problem.

Human papillomavirus (HPV) is a common spherical DNA virus that infects the squamous epithelium of human skin and mucous membranes, and it is the main cause of precancerous lesions of the cervix [6]. Currently, there is a consensus on the prevention of cervical cancer through early HPV vaccination among women. However, misinformation about HPV vaccines on traditional and social media has fostered public concerns regarding their safety [7,8]. According to a recent study, safety concerns are the primary reason parents lack the willingness to allow their children to receive HPV vaccination [9]. With the accumulation of data since the launch of HPV vaccines, many researchers have conducted a series of safety assessment studies on these vaccines [10–12]. However, in general, there are no reports of large-scale serious AEs that affect the safety of HPV vaccines, and in-depth research using large-scale data is lacking.

This study addresses CCA prevention from two perspectives: population-level epidemiology and real-world vaccine safety. Using data from the Global Burden of Disease (GBD) 2021 study, we characterized long-term trends and future projections of CCA burden among women worldwide, thereby highlighting the ongoing public health need for effective preventive strategies. In parallel, we analyzed adverse event reports related to HPV vaccination using the Vaccine Adverse Event Reporting System (VAERS) to describe post-marketing safety reporting patterns that may influence public perception and vaccine acceptance. By integrating these data sources, this study provides a broader public health context for CCA prevention policy.

## Materials and methods

### Data sources

We extracted data on the prevalence, incidence, deaths, disability-adjusted life years (DALYs), and risk factors for CCA among women in 21 regions and 204 countries worldwide from 1990 to 2021 included in the GBD 2021 (accessed March 15, 2025). The GBD 2021 study estimated the burden of 371 diseases and injuries, 288 causes of death, and 88 risk factors from 1990 to 2021 in 204 countries and territories and 811 subnational locations [13]. The database aggregates statistical data and contains no personally identifiable information. Reports of AEs associated with three common HPV vaccines, namely, Cervarix, Gardasil and Gardasil 9, in females from January 2006 to April 2025 were extracted from the VAERS database [14] (accessed March 17, 2025). The VAERS database is used to identify potential safety problems associated with authorized vaccines and does not record sensitive information (name, ID number, etc.) of individuals. VAERS data were analyzed for pharmacovigilance signal detection (disproportional reporting) rather than causal inference. VAERS uses the Medical Dictionary for Regulatory Activities (MedDRA) preferred terms (PT) to code the symptoms of AEs [15]. Descriptive statistics were calculated for the collected reports on the basis of patient age, type of vaccine administered, time to onset of AEs, year of report, and outcome category. In this study, a secondary analysis of deidentified data from the GBD study and the VAERS database was conducted. Therefore, ethical approval and informed consent were not needed.

### Disease burden analysis

The disease burden of CCA was assessed using data from the GBD 2021 study. Age-standardized prevalence, incidence, death and DALY rates were calculated using the WHO standard population to enable comparisons over time. World maps were generated using country boundary data from Natural Earth. To examine long-term temporal patterns and disentangle the effects of age, period, and birth cohort, age–period–cohort (APC) analyses [16] were performed using the National Cancer Institute (NCI) Age–Period–Cohort web tool (https://analysistools.cancer.gov/apc/) [17]. Age, period, and cohort groupings were defined based on the structure of the GBD 2021 dataset and established epidemiological conventions. Age-specific analyses were conducted using 5-year age intervals starting from 15–19 years to ensure sufficient case counts and to satisfy the equal-interval requirement of age–period–cohort modeling [18]. These cut-offs align with prior APC studies using GBD data, ensuring comparability across regions and over time while minimizing random fluctuations in sparsely populated age–period cells. The APC results were summarized using net drift, local drift, age-specific effects, period-specific effects, and cohort-specific effects. Sensitivity analyses for the APC models were conducted by applying alternative identifiability constraints and reference categories, and the resulting age, period, and cohort patterns were compared with those from the primary analysis.

To project future trends in CCA incidence and death, APC projections were further conducted using a Bayesian age–period–cohort (BAPC) framework. Age-specific case counts and corresponding population data were obtained from the GBD 2021 study. To evaluate the predictive performance, retrospective validation was performed by withholding the most recent five years of observed data (2017–2021). Models were fitted using data from earlier years and used to predict age-standardized incidence and death rates for the holdout period. The predictive accuracy was assessed using the mean absolute error (MAE), root mean square error (RMSE) and mean absolute percentage error (MAPE). In addition, the BAPC model incorporates the integrated nested Laplace approximation (INLA) framework [19], which has a lower computational density than traditional Markov Chain Monte Carlo (MCMC) models. Sensitivity analyses for the BAPC models were conducted by modifying key smoothing assumptions, including the use of second-order difference priors, and projections under alternative specifications were compared with those of the primary analysis.

### Disproportionality analysis

To ensure that all the relevant studies on HPV were extracted from the database, we used the NCBI MESH to identify and organize the names of all the HPV vaccines and used four commonly disproportionality algorithms,namely, reporting odds ratio (ROR), proportional reporting ratio (PRR), Bayesian confidence propagation neural network [20] (BCPN), and multi-item gamma Poisson shrinker (MGPS) (S1 Table). The ROR and PRR are sensitive screening tools. An ROR or PRR > 1 suggests a disproportionate association. The BCPNN calculates the information component (IC), which measures the strength of the drug–AE association while stabilizing the estimates for small counts. A positive IC indicates a signal. The MGPS computes the empirical Bayesian geometric mean (EBGM), a shrunken estimate of the PRR. An EBGM > 1 indicates disproportionate reporting. A signal was considered significant only if the lower limit of the 95% confidence interval (CI) for all four algorithms exceeded their respective thresholds, with multiple testing correction applied using the Benjamini-Hochberg procedure to control the false discovery rate (FDR) at 5% [21]. This requirement of concordance across all four algorithms represents a conservative signal-filtering strategy that prioritizes specificity over sensitivity, thereby reducing the likelihood of false-positive signals. We also used hierarchical logistic regression analysis, dividing the data into different groups according to age and vaccine type. As VAERS is a passive spontaneous reporting system, reporting bias, confounding (e.g., underlying conditions), and co-administration of other vaccines or medications cannot be reliably measured or controlled. Therefore, the disproportionality metrics and the stratified regression analyses in this study were intended to characterize disproportional reporting patterns across subgroups rather than to estimate causal effects or incidence risks.

### Designated medical event (DME) list screening

To assess serious and special safety events related to HPV, we conducted a comparative analysis using the DME list of the European Medicines Agency (EMA) to identify valuable positive signals [22]. DMEs were developed on the basis of preferred terms of MedDRA and serves as a crucial tool in pharmacovigilance to prioritize the review of serious AE reports. This ensured that certain reports of serious AEs were not overlooked, even if they did not reach statistical significance.

### Google Trends analysis

We used Google Trends to descriptively explore the changing patterns of public search interest related to HPV and HPV vaccines over time [23]. Google Trends is an online tool that provides standardized search volume indices (ranging from 0 to 100), with the most popular words receiving 100 points; words with half the popularity receiving 50 points; and words with insufficient data receiving 0 points. These indices reflect the relative popularity of specific search terms over time in a specific geographical area [24]. In this study, we obtained the global search trends for the “HPV” and “HPV vaccine” keywords from June 2006 to March 2025 and annual search interest was represented by the mean of monthly Google Trends indices, which were consistent with the time range of the statistics in the VAERS database. This analysis is not intended to test statistical associations between search interest and VAERS reporting patterns, which is descriptive and exploratory in nature.

### Weibull distribution analysis

The Weibull distribution test was used to determine the proportion of changes in the incidence of AEs. The test is a continuous probability distribution whose probability density function and cumulative distribution function can be defined by the scale (α) and shape parameters (β) [25, 26]. The Weibull distribution test was used to analyze the incidence trends of AEs using scale (α) and shape (β) parameters. A shape parameter β < 1 with 95% CI < 1 indicates decreasing AE incidence (early failure pattern), β ≈ 1 with 95% CI including 1 reflects constant occurrence (random failure pattern), β > 1 with 95% CI excluding 1 suggests increasing incidence (wear-out failure pattern). This parameter interpretation categorizes failure dynamics through the distribution’s characteristic curves.

### Sensitivity analysis

Requiring concordance across four disproportional methods is a conservative strategy that prioritizes specificity over sensitivity; therefore, some true but weaker signals may not be captured. To test the robustness of the primary findings, we added a sensitivity analysis. If a signal was marked as a valid signal by the three algorithms, namely, ROR, BCPNN and MGPS, it was regarded as a positive signal. The purpose was to check whether the key signals would still appear even under a less stringent rule, thus strengthening confidence in the present results if the signals remain consistent.

### Statistics

All the graphs and statistical analyses were performed using the BAPC (version 0.0.36), INLA (version 23.04.24), and ggplot2 (version 3.4.4) packages in R Studio software (version 4.2.2). P < 0.05 (two-tailed) was considered to indicate statistical significance.

## Results

### Global burden of CCA

In 2021, 3.4 million prevalent cases of CCA were recorded globally, with an age-standardized point prevalence (ASPR) of 79.3 per 100,000 women, representing a 1.5% increase since 1990. There were 0.67 million new cases of CCA globally, with an age-standardized incidence rate (ASIR) of 15.3 per 100,000 women —a −15.4%decrease since 1990. CCA has resulted in 300,000 deaths, with an age-standardized rate (ASR) of 6.6 per 100,000 women, representing a −31.6% reduction since 1990. The global number of DALYs for CCA in 2021 was 9.9 million, with an ASR of 226.3 DALYs per 100,000 women, representing a −31.5% decrease since 1990.

### Regional burden of CCA

In 2021, the ASPR was highest in Andean Latin America (170.2), and lowest in Western Europe (59.0). The ASIR peaked in southern sub-Saharan Africa (42.4) but was minimal in North Africa and the Middle East (4.7). Southern sub-Saharan Africa had the highest age-standardized death rate (ASDR) (23.9), while South Asia reported the most deaths (0.07 million). Australasia had the lowest number of deaths (500 cases, ASDR 1.8). DALY rates were highest in central sub-Saharan Africa (813.6), and lowest in Australasia (61.7).

From 1990 to 2021, the ASPR increased the most in East Asia (67.9%), but declined sharply in Australasia (−44%). The ASIR increased the most in southern sub-Saharan Africa (41.9%) but decreased the most in Australasia (−50.5%). All regions except southern sub-Saharan Africa experienced reduced ASDR and DALY rates (maximum decreases of −37.1% and −31.4%, respectively). East, South, and Southeast Asia had the highest 2021 burden across all the metrics.

### National burden of CCA

**Fig 1:**
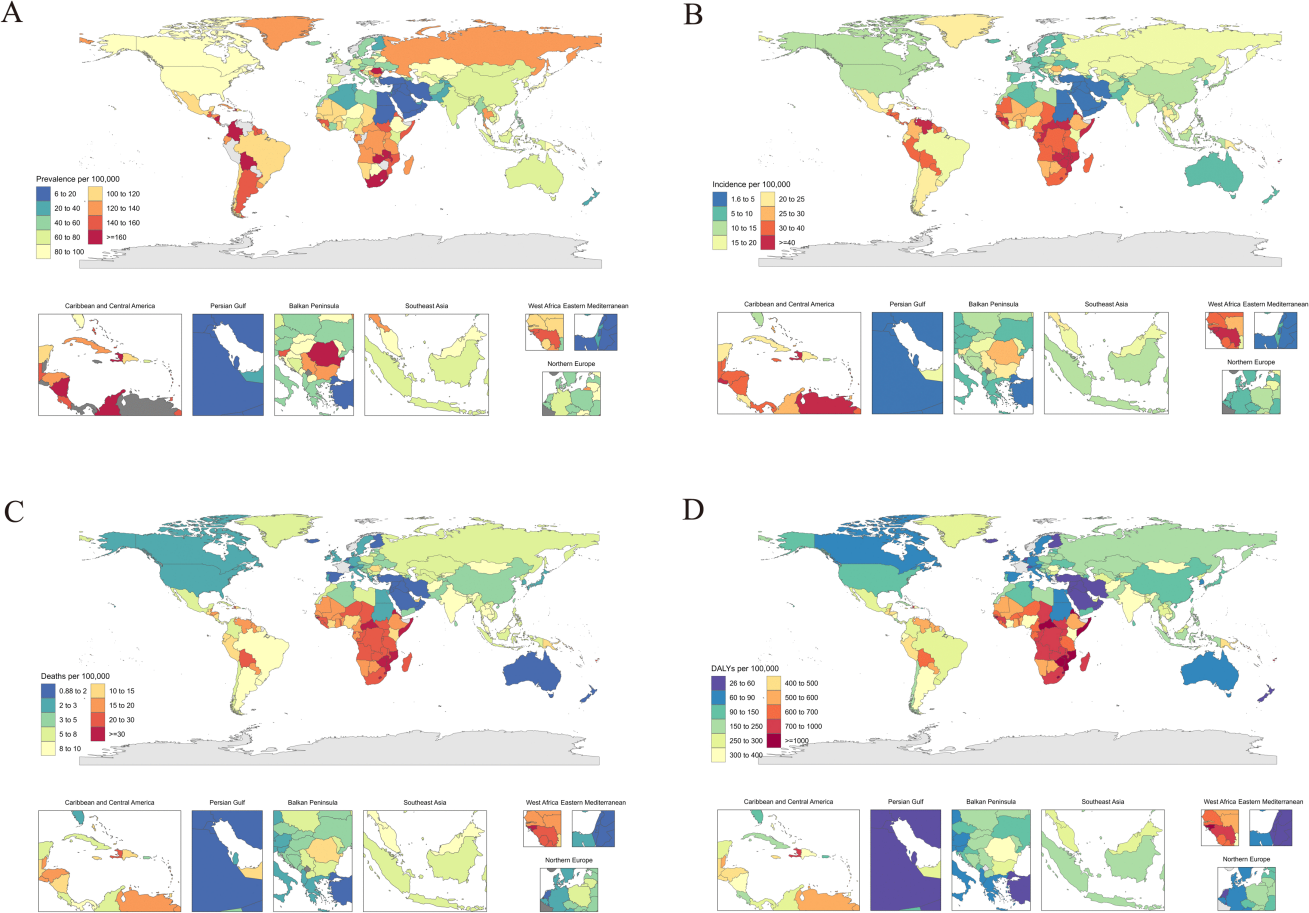
Age-standardized rates of CCA per 100000 women by country. **(A)** Age-standardized point prevalence of CCA per 100,000 people in 2021, by country. **(B)** Age-standardized incidence rate of CCA per 100, 000 people in 2021, by country. **(C)** Age-standardized deaths rate of CCA per 100,000 people in 2021, by country. **(D)** Age-standardized DALY rate of CCA per 100,000 people in 2021, by country. Base map source: Natural Earth (public domain, CC0).

In 2021, CCA burden varied significantly across nations. Venezuela (279.6) had the highest ASPR, while Palestine (6.3) reported the lowest. ASIR peaked in Kiribati (70.0/100,000) and Lesotho (60.8), whereas minimal rates were detected in Palestine (1.7) and Kuwait (2.1). The ASDR was highest in Kiribati (45.1 deaths/100,000) and lowest in Saudi Arabia (0.89), with DALY rates exceeding 1400/100,000 in Kiribati but below 30 in Kuwait and San Marino (Fig 1).

From 1990 to 2021, Lesotho experienced the steepest increases in the ASPR (147.1%), ASIR (136.3%), and ASDR (123.5%), whereas Kuwait exhibited marked decreases (ASPR: −72.5%; ASIR: −75.4%; ASDR: −79%). Italy and Egypt also experienced increasing trends, whereas New Zealand and the Maldives showed substantial decreases across metrics. Only 13 countries experienced increases in ASDRs, led by Lesotho and Zimbabwe, while 15 countries reported higher DALYs, including Eswatini. Conversely, Kuwait, Maldives, and New Zealand achieved the greatest DALY reductions (−74% to −80.8%).

### Association of CCA with different age groups

**Fig 2:**
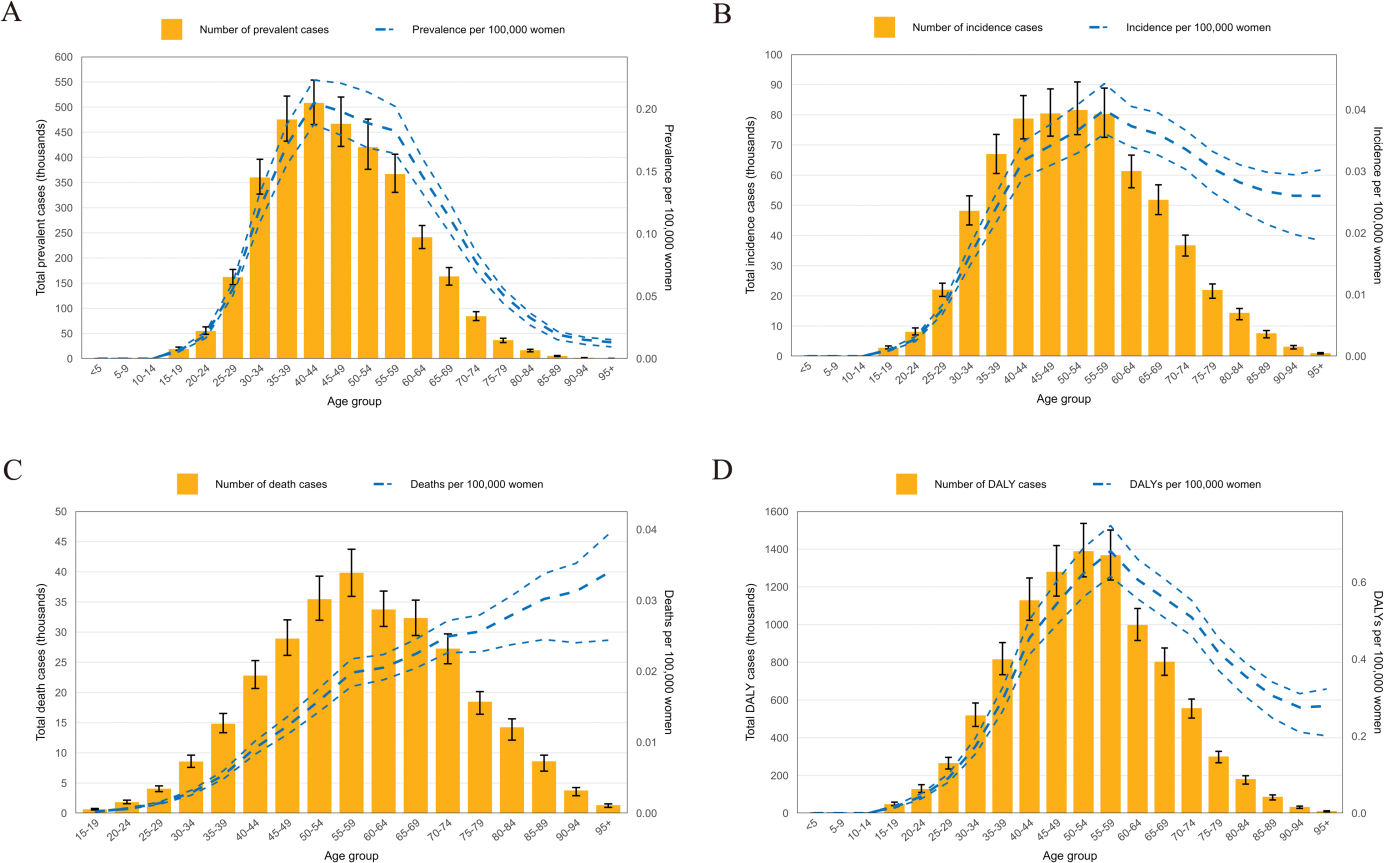
Age-standardized rates trends of CCA per 100,000 women in different age groups. **(A)** Global number of prevalent cases and prevalence of CCA per 100,000 women, by age in 2021. **(B)** Global number of incident cases and incidence of CCA per 100, 000 women, by age in 2021. **(C)** Global number of deaths and deaths rate of CCA per 100,000 women, by age in 2021. **(D)** Global number of DALYs and DALY rate of CCA per 100,000 women, by age in 2021. Lines indicate cases with 95% uncertainty intervals; dotted and dashed lines indicate 95% upper and lower uncertainty intervals.

By analyzing the number of affected women of different ages, we detected an association resembling a reversed “V” between the global point prevalence of CCA and the age of onset. The global point prevalence of CCA in 2021 started to increase among individuals aged 15–19 years, peaking in those aged 40–44 years. Similarly, the 40–44 age group had the most prevalent cases, which decreased as age increased (Fig 2A). The incidence rate of CCA reached its peak in the 55–59 age group (Fig 2B). The global CCA death rate was the highest in the oldest age group (>95 years). The number of deaths caused by CCA was the highest in the 55–59 age group, and then gradually decreased as age increased (Fig 2C). Similar to the incidence results, the DALY rate was also the highest in the 55–59 age group, and the highest number of cases was in the 50–54 age group (Fig 2D).

### Association of CCA with the sociodemographic index (SDI)

**Fig 3:**
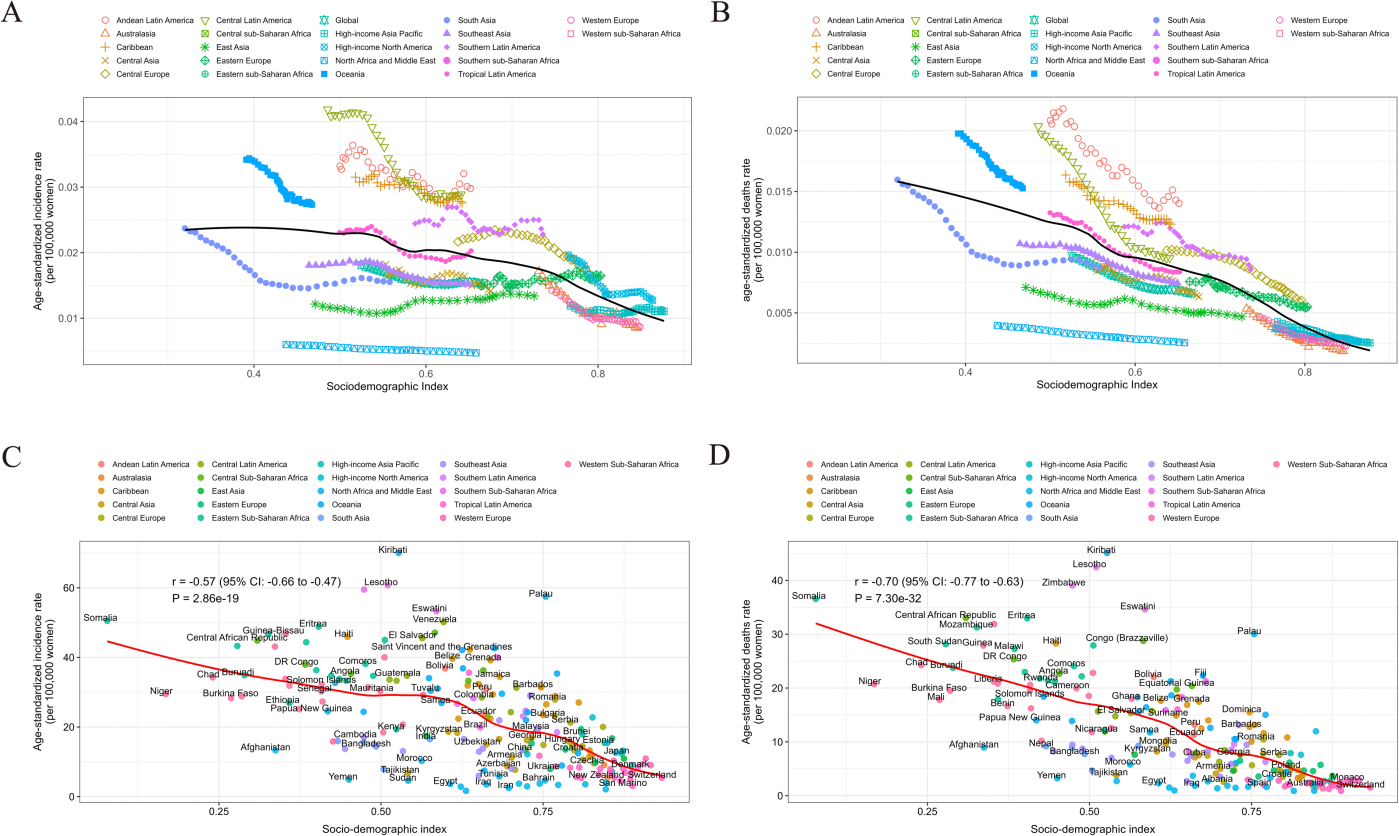
ASIR/ASDR of CCA for 21 regions and 204 countries and territories, by SDI, in 1990–2021. **(A)** ASIR of CCA for 21 regions, by SDI, in 2021. **(B)** ASDR of CCA for 21 regions, by SDI, in 2021. Thirty points are plotted for each region and show the observed ASIR/ASDR from 1990 to 2021 for that region. **(C)** ASIR of CCA for 204 countries and territories, by SDI, in 2021. **(D)** ASDR of CCA for 204 countries and territories, by SDI, in 2021. Each point shows the observed ASIR/ASDR for each country in 2021. Black solid line shows the expected values based on SDI and disease rates in all locations. Regions or countries above the solid line represent a higher-than-expected burden and regions below the line show a lower-than-expected burden.

At the regional level, there was a negative correlation between the SDI and ASIR/ASDR from 1990 to 2021. The ASIR/ASDR decreased with increasing SDI. Oceania, Andean Latin America, and Central Latin America had higher-than-expected incidence and death rates on the basis of their SDI. In contrast, North Africa and the Middle East, East Asia, and Southeast Asia experienced lower burdens than anticipated from 1990 to 2021 (Fig 3A and 3B). At the national level, in 2021, the ASIR/ASDR burden of CCA decreased with increasing socioeconomic development (ASIR: r = −0.57, P = 2.86e-19, ASDR: r = −0.70, P = 7.30e-32). Kiribati, Lesotho, and Zimbabwe experienced unexpectedly high burdens, whereas Palestine, Egypt, and Sudan experienced much lower-than-expected burdens (Fig 3C and 3D).

### APC effect analysis

**Fig 4:**
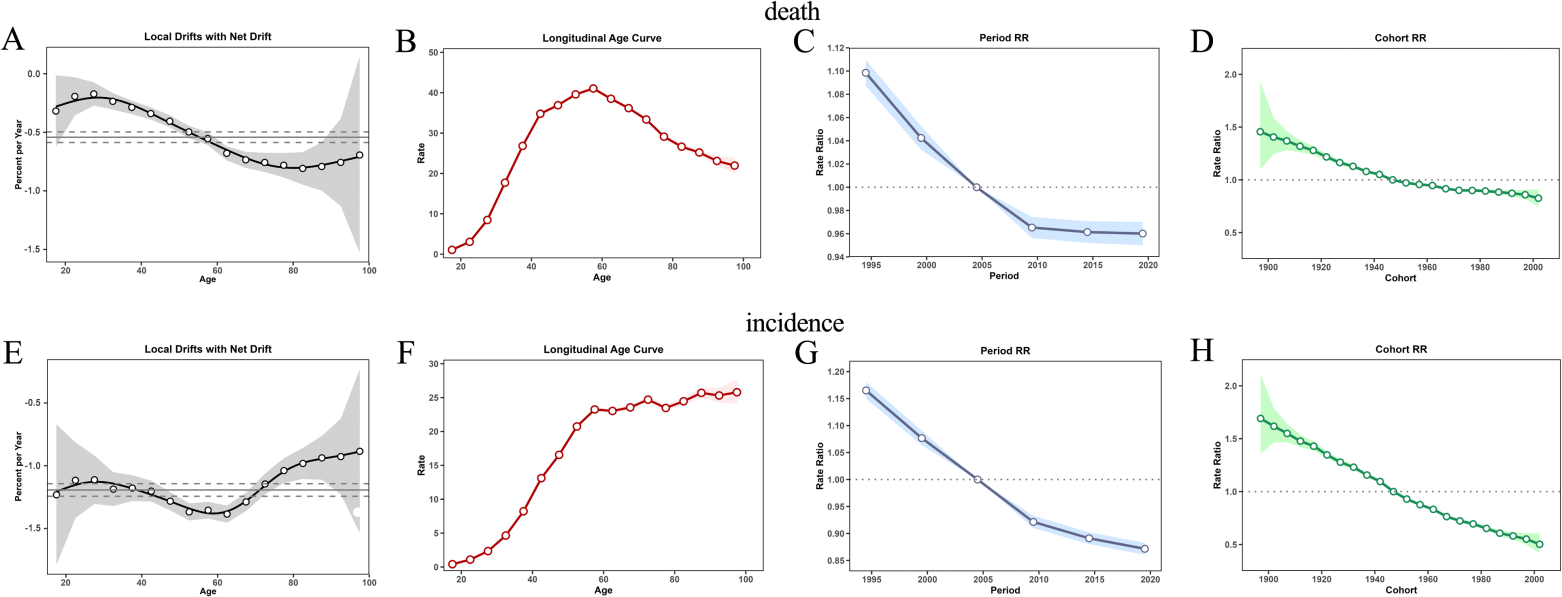
Results of age–period–cohort (APC) model analysis. **(A)** Annual percentage changes of the ASDR (net drift) and estimated age-specific local drifts. **(B)** Longitudinal age curves of female CCA deaths rates (per 100,000) adjusted for period effects. **(C)** Period rate ratios for death: adjusted for age and non-linear cohort effects by year. **(D)** Cohort rate ratios for death: adjusted for age and non-linear period effects. **(E)** Annual percentage changes of the ASIR (net drift) and estimated age-specific local drifts. **(F)** Longitudinal age curves of female CCA incidence rates (per 100,000) adjusted for period effects. **(G)** Period rate ratios for incidence: adjusted for age and non-linear cohort effects by year. **(H)** Cohort rate ratios for incidence: adjusted for age and non-linear period effects. RR > 1 indicates that the relative risk of death/incidence is higher for this birth period/cohort compared to the reference period/cohort. RR < 1 implies that the relative risk of death/incidence is lower for this birth period/cohort compared to the reference period/cohort.

APC analysis revealed a global net drift of −1.19% per year for the CCA death rate, indicating an overall decline. However, local drift was associated with decreasing death rates among individuals younger than 67.5 years but increasing rates among individuals older than 72.5 years (P = 4.53e-06). Age effects demonstrated increasing death rate with age within birth cohorts. Period effects reflected lower death rates in recent years, whereas cohort effects highlighted higher death risk in those born before 1947, which declined thereafter (Fig 4A–4D).

For the incidence rate, the net drift was −0.54% per year. Local drift revealed increasing incidence in individuals younger than 57.5 years but decreasing incidence in individuals older than 62.5 years (P = 3.52e-25). Age effects revealed increasing incidence in individuals younger than 57.5 years, but reversed reversing in older groups. Period effects indicated sustained declines post-2005, and cohort effects mirrored death rate trends, with a higher risk in pre-1947 birth cohorts (Fig 4E–4H). The overall age, period, and cohort patterns remained consistent across alternative APC model specifications.

### Prediction of CCA burden in females

**Fig 5:**
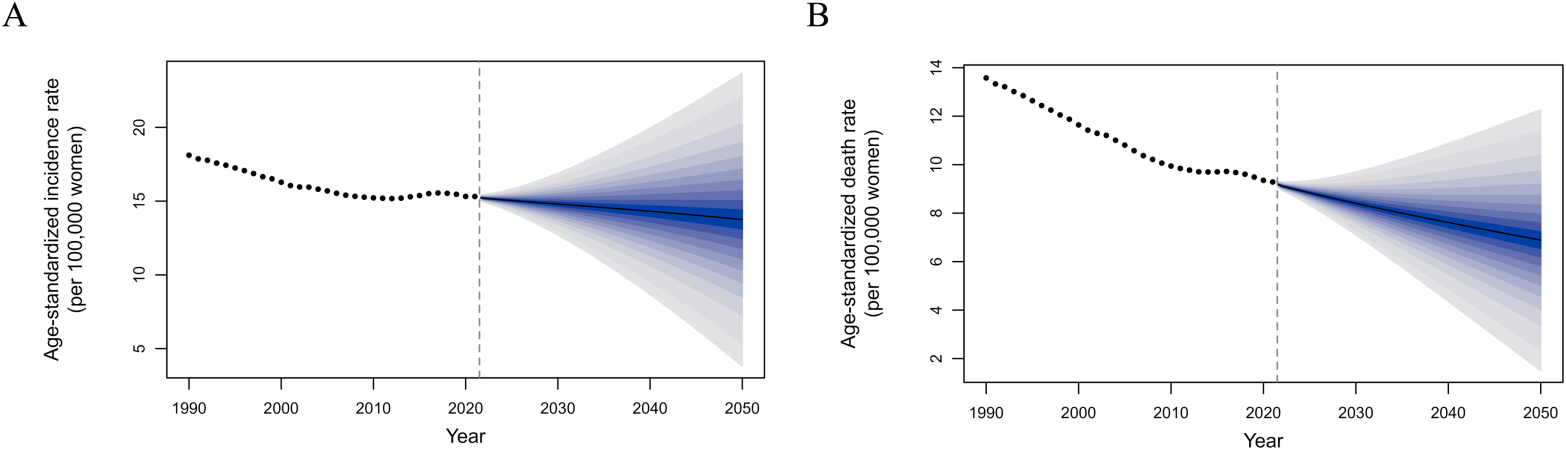
Bayesian age–period–cohort (BAPC) model–based projections of CCA. **(A)** ASIR of HPV infection in women, 1990–2050. **(B)** ASDR attributable to HPV infection in women, 1990–2050.

The incidence and death rates of CCA in women were predicted using the BAPC model. As shown in Fig 5A, the worldwide ASIR for CCA is expected to gradually decline over the next 30 years. It is estimated that by 2050, the ASIR for CCA in women will be 13.74 cases per 100,000, a decrease of −10.25% compared with the rate in 2021. We estimated the ASDR for CCA from 2022 to 2050 (original data starting from 15 years of age) (Fig 5B). By 2050, the ASDR of CCA in women is expected to be 6.88 cases per 100,000, a decrease of −25.85% compared with the ASDR of CCA in 2021. The United Nations World Population Prospects data predict 656,603 new cases of CCA and 328,798 related deaths worldwide by 2050, suggesting that the situation will remain serious.

Retrospective validation demonstrated good predictive performance for the ASIR. For the 2017–2021 holdout period, the predicted incidence closely matched the observed values, with an MAE of 0.24 per 100,000 women, an RMSE of 0.30, and a MAPE of 1.56%, indicating high agreement between the predicted and observed trends. In contrast, death projections showed larger deviation in level during validation. For the ASDR, the MAE and RMSE were approximately 2.8 per 100,000 women, with a higher MAPE reflecting low baseline death rates and greater year-to-year variability. Importantly, the death models consistently captured the overall declining trend across different model specifications, including alternative smoothing assumptions, supporting the robustness of the trend estimates.

### Descriptive overview of HPV vaccination reports in the VAERS

In low-SDI regions, the persistent burden of CCA underscores the critical need for effective primary prevention, including HPV vaccination. However, the successful implementation of vaccination programs not only depends on the availability of vaccines, but also on the public’s acceptance and trust in vaccine safety. To complement the results of epidemiological assessment, we analyzed VAERS reports related to HPV vaccination to clarify the characteristics of the reported AEs and detect potential biased reporting signals. A total of 41,731 reports on HPV vaccination in females were selected from the VAERS database, corresponding to 210,347 AEs. In terms of age distribution, vaccination reports among children aged 9–17 years accounted for the greatest proportion (65.04%) (Table 1). Approximately half of the participants experienced AEs on the day of vaccination, and most of the remaining participants developed symptoms within 1 month, with an average onset time of 39.5 days. The incidence of AEs gradually decreased over time (S1A Fig). In terms of year distribution, the number of vaccine reports peaked in the first 2 years after its launch, with 5,750 cases in 2007 and 6,724 cases in 2008, and this number subsequently decreased to < 4,000 cases per year and remained stable (S1B Fig).

**Table 1:** Characteristics of VAERS reports of HPV vaccines in women, 2006–2025.

	0-8 years	9-17 years	18-26 years	27-35 years	36-45 years	>45 years	Overall
	(N = 316)	(N = 27141)	(N = 11601)	(N = 1561)	(N = 867)	(N = 245)	(N = 41731)
Age years							
Mean (SD)	3.03 (2.80)	13.8 (2.05)	21.3 (2.67)	30.4 (2.75)	39.8 (2.76)	55.3 (9.35)	17.2 (6.73)
Median [Min, Max]	1.88 [0, 8.00]	14.0 [9.00, 17.0]	21.0 [18.0, 26.0]	30.0 [27.0, 35.0]	40.0 [36.0, 45.0]	52.0 [46.0, 97.0]	16.0 [0, 97.0]
Time to onset							
Mean (SD)	49.4 (356)	37.5 (227)	40.9 (230)	60.7 (295)	45.3 (585)	45.5 (393)	39.5 (245)
Serious							
N	299 (94.6%)	21633 (79.7%)	9825 (84.7%)	1173 (75.1%)	620 (71.5%)	214 (87.3%)	33764 (80.9%)
Y	17 (5.4%)	5508 (20.3%)	1776 (15.3%)	388 (24.9%)	247 (28.5%)	31 (12.7%)	7967 (19.1%)
AE Report Number	788	141190	53112	8842	5474	941	210347
Vaccine Name							
HPV (CERVARIX)	6 (1.9%)	2787 (10.2%)	352 (3.0%)	120 (7.7%)	80 (9.2%)	20 (8.0%)	3365 (8.0%)
HPV (GARDASIL 9)	188 (59.1%)	5748 (21.1%)	1908 (16.4%)	561 (35.8%)	437 (50.1%)	136 (54.6%)	8978 (21.5%)
HPV (GARDASIL)	124 (39.0%)	18688 (68.6%)	9365 (80.6%)	884 (56.5%)	355 (40.7%)	93 (37.3%)	29509 (70.5%)

### Statistics of safety signals

We calculated the HPV vaccination signal at the System Organ Class (SOC) level which revealed that 27 SOCs were implicated in HPV vaccine-related AEs, with Nervous system disorders showing the highest incidence rate (19.8%). Notably, four SOCs that conformed to the criteria set by the four disproportionality algorithms included Pregnancy, puerperium and perinatal conditions (ROR:3.58, 95%CI: 3.38–3.80, P < 0.0001), Neoplasms benign, malignant and unspecified (incl cysts and polyps) (ROR:3.21, 95%CI: 2.95–3.48, P = 1.30e-190), Endocrine disorders (ROR:2.41, 95%CI: 2.17–2.68, P = 7.58e-62) and Congenital, familial and genetic disorders (ROR:2.91, 95%CI: 2.47–3.43, P = 2.03e-40).

**Fig 6:**
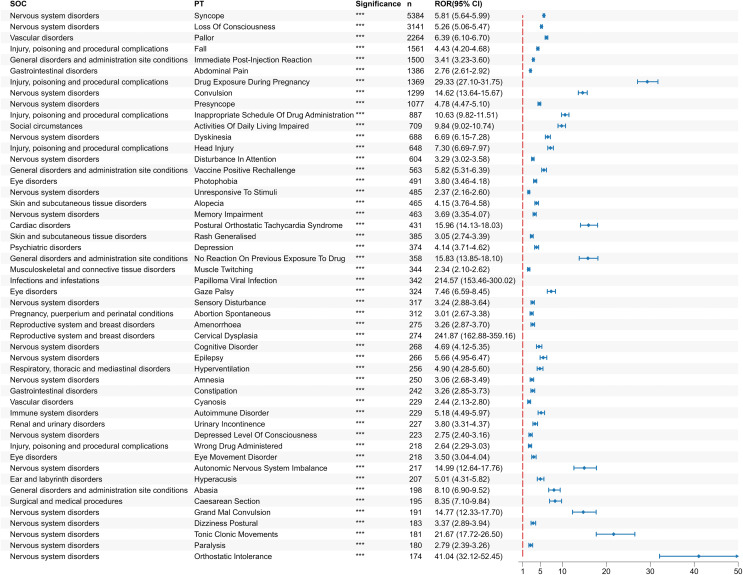
Screening of safety signals for HPV vaccination. Forest plot of PTs with a top 50 cases. The blue arrows indicate that the 95% confidence interval’s lower limit for ROR is above 50.

At the PT level, 6359 PTs were included in the follow-up analysis after categories such as social circumstances, COVID-19, product issues, and other unrelated PTs were excluded. Among them, 1102 positive signals simultaneously conformed to all four algorithms. After controlling for the false discovery rate (FDR < 0.05), the statistical significance remained unchanged (P < 0.05). The three most frequently reported symptoms were Syncope (ROR = 5.81, 95%CI:5.64–5.99, P < 0.0001), Loss of consciousness (ROR = 5.26, 95%CI: 5.06–5.47, P < 0.0001) and Pallor (ROR = 6.39, 95%CI: 6.10–6.70, P < 0.0001).This finding is consistent with the results of previous clinical trials. The top three PT signals with the highest ROR ratios are Cervical conisation (ROR = 809.56, 95%CI: 198.41–3303.25, P < 0.0001), Vaginitis bacterial (ROR = 714.19, 95%CI: 97.39–5237.25, P = 1.48e-145), and Cervix carcinoma stage 0 (ROR = 618.95, 95%CI: 83.99–4561.33, P = 5.88e-125). In addition, some significant AEs not previously anticipated were identified, including Visual disturbance (n = 67, ROR = 16.62,95%CI:12.16–22.70, P = 9.42e-125) and Hyperacusis (n = 207, ROR = 5.01,95%CI:4.31–5.82, P = 1.71e-119). A forest plot of the top 50 reported PTs is shown in Fig 6.

### DME list screening

Among the 1102 positive signals, the following six signals corresponded to the DME list: Blindness (ROR = 2.60, 95%CI:2.20–3.07), Pancreatitis (ROR = 3.22, 95%CI:2.26–4.58), Aplastic anaemia (ROR = 6.74, 95%CI:3.94–11.56), Hepatic failure (ROR = 3.66, 95%CI:2.09–6.43), Deafness transitory (ROR = 5.04, 95%CI:2.63–9.65) and Hepatic necrosis (ROR = 7.32, 95%CI: 2.39–22.46). These signals were categorized as Eye disorders, Gastrointestinal disorders, Hepatobiliary disorders, Blood and lymphatic system disorders, Ear and labyrinth disorders at the SOC level. Notably, the highest ROR value was observed for Hepatic necrosis (7.32), while the number of cases of Blindness was the highest (155).

### Internet search interest and VAERS reporting

**Fig 7:**
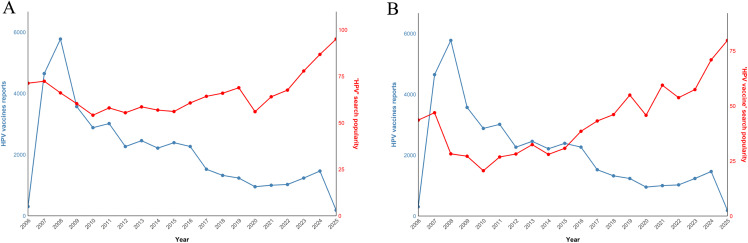
Analysis of HPV Vaccine Reports and Google Search Trends. **(A)** Number of HPV vaccine reports of VAERS and search popularity of the “HPV” topic on Google Trends. **(B)** Number of HPV vaccine reports of VAERS and search popularity of the “HPV vaccine” topic on Google Trends.

Global Google Trends data revealed that the public search interest for the “HPV” and “HPV vaccine” keywords changed over time (Fig 7A and 7B). After the launch of the HPV vaccine, the search interest gradually increased and reached an early peak but then declined and gradually stabilized. Around 2016, the search interest showed a rising trend again. During the same period, the number of annual reports submitted to VAERS related to the HPV vaccine exhibited different time patterns, the number of reports was higher in the years following the vaccine launch, and then lower and more stable thereafter. The fluctuations in search interest are in line with the fluctuations in the number of reports.

### Weibull distribution analysis

To further substantiate the temporal patterns observed in the risk of HPV vaccine-related AEs, a Weibull distribution analysis was conducted. From the overall analysis, a shape parameter (β) of 0.446 was derived, with a 95% CI of 0.44–0.45, indicating an early failure-type model in which the rate of AEs decreased over time.

### Age subgroup analysis

**Fig 8:**
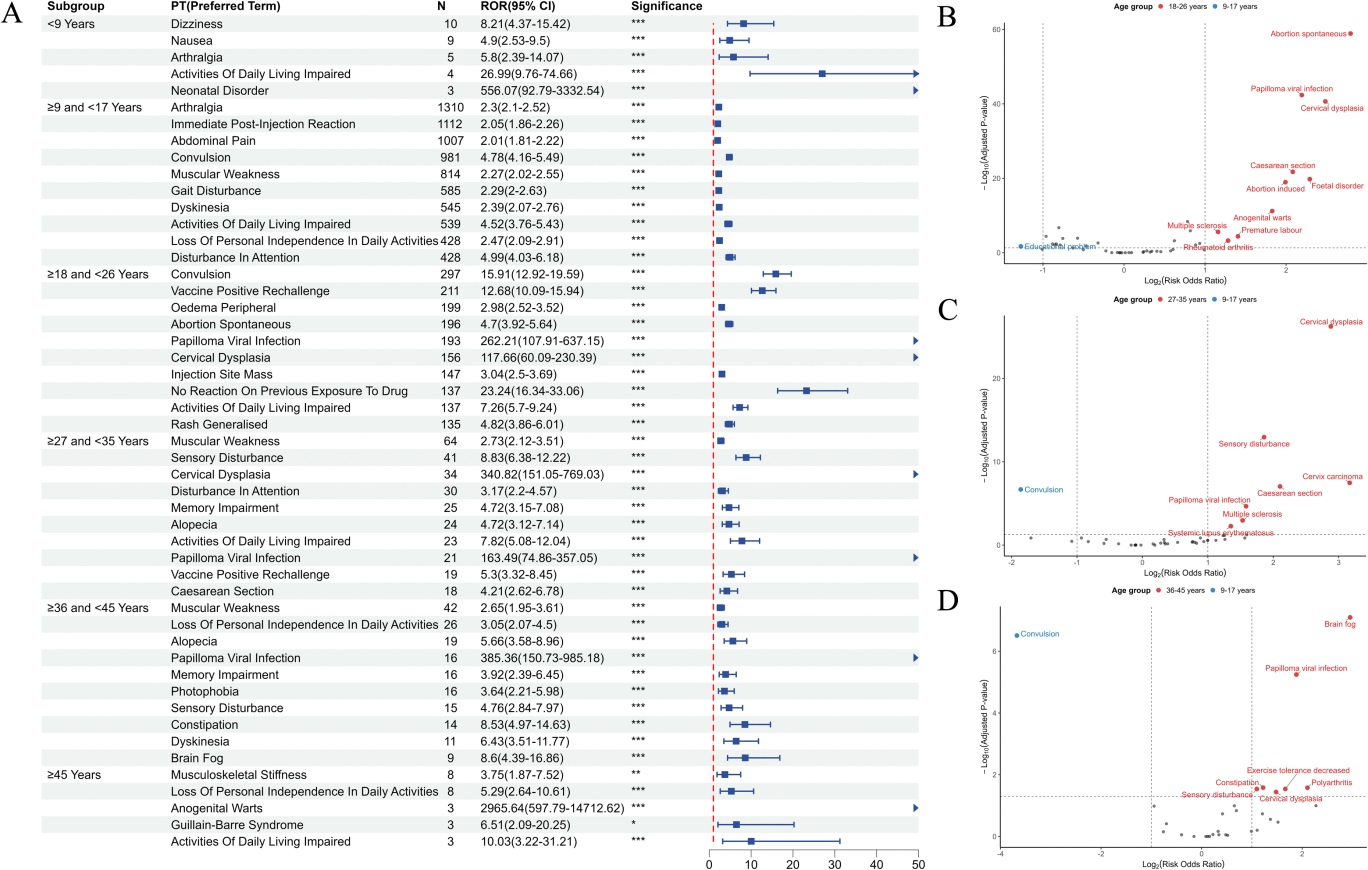
Safety signals for vaccine administration differ by age. **(A)** Forest plot of age-differentiated PT risk signals. **(B)** Volcano plot of different AEs between 9–17 years group and 18–26 years group receiving HPV vaccine injections. **(C)** Volcano plot of different AEs between 9–17 years group and 27–35 years group receiving HPV vaccine injections. (**D)** Volcano plot of different AEs between 9–17 years group and 36–45 years group receiving HPV vaccine injections. The horizontal coordinate shows the log2 ROR value, and the vertical coordinate indicates the adjusted p-value after log10 conversion. Significant signals are highlighted and annotated in prominent colors. P-values were adjusted using the Bonferroni correction method.

To analyze the relationship between age and HPV vaccine-related AEs, we categorized the studies into the following six different age groups: < 9, 9–17, 18–26, 27–35, 36–45, and > 45 years. In the < 9 year group, five positive signals were selected through screening. The top 3 PTs were Dizziness (ROR = 8.21, 95%CI:4.37–15.42, P = 9.71e-13), Nausea (ROR = 4.90, 95%CI:2.53–9.50, P = 4.69e-06), and Arthralgia (ROR = 5.80, 95%CI:2.39–14.07, P = 0.0003). For 9–17 years, Arthralgia (ROR = 2.30, 95%CI:2.10–2.52, P = 1.14e-71), Immediate post-injection reaction (ROR = 2.05, 95%CI:1.86–2.26, P = 4.57e-48), and Abdominal pain (ROR = 2.01, 95%CI:1.81–2.22, P = 2.78e-41) were predominant among 169 positive signals. A total of 80 positive signals found in the 18–26 year group. Reports of Convulsion (ROR = 15.91, 95%CI:12.92–19.59, P = 5.10e-267), Vaccine-positive rechallenge (ROR = 12.68, 95%CI:10.09–15.94, P = 4.40e-170), and Oedema peripheral (ROR = 2.98, 95%CI: 2.52–3.52, P = 4.05e-39) had the highest number. For 27–35 years, the top three PTs among the 102 positive signals were Muscular weakness (ROR = 2.73, 95%CI: 2.12–3.51, P = 1.91e-14), Sensory disturbance (ROR = 8.83, 95%CI: 6.38–12.22, P = 4.89e-53), and Cervical dysplasia(ROR = 340.82, 95%CI:151.05–769.03, P < 0.0001). For the 36–45 year age group, 75 positive signals were identified, with Muscular weakness (ROR = 2.65, 95%CI:1.95–3.61, P = 2.93e-09), Loss of independence (ROR = 3.05, 95%CI:2.07–4.50, P = 7.66e-08), and Alopecia (ROR = 5.66, 95%CI: 3.58–8.96, P = 1.52e-14) ranking among the top three in terms of quantity. In the > 45 year age group, Musculoskeletal stiffness (ROR = 3.75, 95%CI:1.87–7.52, P = 0.0011), Loss of independence (ROR = 5.29, 95%CI:2.64–10.61, P = 7.78e-06), and Anogenital warts (ROR = 2965.64, 95%CI: 597.79–14712.62, P < 0.0001) were among the top 3 among 5 positive signals (Fig 8A).

We also analyzed the differences in AE signals after HPV vaccination among the different age groups using volcano plots. The screening threshold was set as adjusted P < 0.05 and |log2(ROR)| > 1, and the AEs with statistical significance were noted. Compared with the 18−26 age group, the 9–17 age group had higher risks of Educational problems (ROR = 8.69, 95%CI:4.32–17.45) (P = 0.02) (Fig 8B), and Convulsion risk (ROR = 4.78, 95%CI:4.16–5.49) was greater in the 9–17 age group than in the 27–35 (P = 2.12e-07) and 36–45 (P = 3.09e-07) age groups (Fig 8C and 8D).

### Brand subgroup analysis

There are certain differences in PT signals among the three different types of vaccines. There were 244, 531 and 188 positive signals for the Cervarix, Gardasil and Gardasil 9 vaccines, respectively. For the Cervarix vaccines, the top three PTs were Syncope (ROR = 4.83, 95%CI:4.44–5.25, P < 0.0001), Loss of consciousness (ROR = 6.71, 95%CI:6.13–7.34, P < 0.0001), and Presyncope (ROR = 14.48, 95%CI:13.05–16.06, P < 0.0001), which all belong to Nervous system disorders at the SOC level. The PT signal with the highest ROR value was School refusal (ROR = 585.76, 95%CI: 208.81–1643.22, P < 0.0001). For the Gardasil and Gardasil 9 vaccines, the top three PTs were Syncope (Gardasil: ROR = 5.32, 95%CI: 5.14–5.51, P < 0.0001; Gardasil 9: ROR = 5.61, 95%CI: 5.27–5.96, P < 0.0001), Loss of consciousness (Gardasil: ROR = 4.09, 95%CI: 3.90–4.29, P < 0.0001; Gardasil 9: ROR = 6.35, 95%CI: 5.90–6.82, P < 0.0001), and Pallor (Gardasil: ROR = 4.69, 95%CI: 4.42–4.97, P < 0.0001; Gardasil9: ROR = 7.78, 95%CI: 7.15–8.46, P < 0.0001). The PT signals with the highest ROR value for Gardasil and Gardasil9 were Carcinoma in situ (ROR = 608.26, 95%CI: 81.2–4556.56, P = 4.20e-118) and Bartonellosis (ROR = 403.73, 95%CI: 41.99–3881.55, P = 2.55e-45). Volcano plot analysis revealed that compared with the Cervarix vaccine, the Gardasil vaccine resulted in more disproportional reporting signals for Papilloma viral infection(P = 9.52e-06), Anogenital warts(P = 0.0055), and Postural orthostatic tachycardia syndrome(P = 0.0026). Compared with the Cervarix vaccine, the Gardasil 9 vaccine exhibited more disproportional signals for Posture abnormality(P = 0.001). (S2A-S2C in S2 Figs). However, owing to the limited number of VAERS reports, these signals need continued attention and further verification.

### Sensitivity analysis

A sensitivity analysis of AEs to the HPV vaccine was conducted to assess the robustness of the main research results. After signals unrelated to adverse reactions were excluded, no new positive signals meeting the FDR correction criteria were detected. Moreover, the key signals discussed in the main text (syncope and loss of consciousness) were still robustly detected under this standard.

## Discussion

This study integrated two complementary but methodologically distinct components: global epidemiological burden estimation and post-marketing vaccine safety surveillance. The GBD 2021 analysis identifies populations and regions with the highest CCA burden, while the VAERS-based assessment provides insight into real-world safety reporting patterns for HPV vaccination, a cornerstone of primary prevention. Although these data sources address different questions and do not permit causal linkage, their combined interpretation provides contextual information to inform prevention strategies and risk communication. The statistical results of the GBD revealed that the number of new CCA cases and related deaths has continued to increase globally over the past 20 years (S3 Fig). CCA is the most common cancer in women in 25 countries and the leading cause of cancer-related deaths in 37 countries [1], posing a major public health threat and a substantial disease burden worldwide.

### Changes in disease burden

Globally, the incidence and death of CCA show substantial geographic heterogeneity. From 1990 to 2021, the ASIR declined in 82% of countries and regions, and the ASDR declined in 94% of countries and regions, particularly in Europe, Oceania, and North America. In contrast, increasing or persistently high trends were observed in several low- and middle-resource settings, including parts of East Asia (e.g., the Democratic People’s Republic of Korea), southern sub-Saharan Africa (e.g., Lesotho), Central and North America (e.g., Guatemala), and Pacific Island nations (e.g., Kiribati). In 2021, the ASIR ranged from 4.7 per 100,000 population in the Middle East and North Africa to 42.4 per 100,000 in southern sub-Saharan Africa, whereas the ASDR ranged from 1.8 per 100,000 in Australasia to 25.1 per 100,000 in central sub-Saharan Africa, representing more than a tenfold regional difference. Kiribati exhibited the highest ASIR (70.0 per 100,000) and ASDR (45.1 per 100,000) worldwide. These findings highlight pronounced global disparities in the burden of CCA as estimated by the GBD 2021. Previous studies have suggested that differences in access to HPV vaccination, CCA screening, and treatment services may contribute to the observed regional patterns, particularly in resource-limited or conflict-affected settings [27,28]. In addition, the GBD 2021 comparative risk assessment identified unsafe sex and smoking as leading contributors to CCA-related deaths and DALYs, providing a partial epidemiological context for these disparities. Age-specific analyses further revealed heterogeneity across regions. In southern sub-Saharan Africa, women younger than 45 years accounted for approximately 41% of all CCA cases in 2021. A younger age at diagnosis was also observed in China, where the average age at diagnosis declined to 44.7 years, compared with earlier periods. While changes in sexual behavior, urbanization, and prevention awareness have been proposed as potential explanations in prior studies, such factors cannot be directly evaluated using GBD data and should therefore be interpreted cautiously. Consistent with these patterns, the ASIR and ASDR demonstrated strong gradients across SDI levels, with low-SDI countries experiencing substantially higher incidence and mortality than high-SDI countries. These gradient underscores the persistent association between socioeconomic development level and the global burden of CCA.

### Vaccine safety assessment

Nearly 20 years have passed since the launch of the world’s first HPV vaccine. Numerous clinical trials have investigated the efficacy and safety of HPV vaccines. The present study used the VAERS database to further evaluate vaccine safety. Across all age groups, the majority of reported AEs following HPV vaccination were nonserious (80.9%) and were clustered in younger recipients, which is consistent with the target population of HPV immunization programs. Age-stratified analyses revealed that reporting patterns differed across age subgroups, with younger age groups contributing a higher volume of reports and older recipients accounting for a smaller proportion of reports with relatively different adverse event profiles. Subgroup analyses by vaccine product indicated heterogeneity in disproportional reporting signals across Cervarix, Gardasil, and Gardasil 9. Across all three vaccine products, Syncope and Loss of consciousness were among the most frequently reported AEs. This pattern is biologically plausible and has been widely reported following vaccination in adolescents and young adults, primarily reflecting vasovagal reactions and anxiety-related responses to injection, rather than vaccine-specific toxicity [29]. These reactions are likely related to transient autonomic responses to injection stress rather than vaccine components. Moreover, syncope and loss of consciousness have also been commonly reported following other injectable vaccines [30], particularly in younger populations [31], and are generally classified as nonserious AEs in post-marketing surveillance systems [32]. The consistent predominance of these events across HPV vaccine products therefore likely reflects shared characteristics of the vaccination process and target population, rather than meaningful differences in product-specific safety profiles. However, these comparisons were based on raw disproportionality metrics derived from VAERS reports and should be interpreted with caution. Differences in reporting patterns across vaccine products may be influenced by variations in vaccine uptake, approval timelines, target age populations, and reporting intensity over time, which are not captured in the VAERS. As a result, the observed differences represent only product-specific reporting patterns within the post-marketing surveillance system. Taken together, the age-stratified and vaccine-specific analyses provide a descriptive overview of HPV vaccine safety reporting patterns and serve to identify signals for further clinical review and epidemiological investigation.

By comparing the present disproportionality results with the EMA DME list, we identified six preferred DME-related terms that showed disproportional reporting signals in the VAERS (Blindness, Pancreatitis, Aplastic anaemia, Hepatic failure, Transient deafness, and Hepatic necrosis). These signals should be interpreted as pharmacovigilance alerts for prioritizing clinical review and further epidemiological verification.

Although HPV mainly affects women, the risk of men contracting HPV-related diseases (such as anal cancer and genital warts) is receiving increasing attention. HPV vaccination not only protects the health of men but also provides indirect protection for women. In 2022, 6% of men in the global target age range completed the first dose of the vaccine and 5% completed all immunization doses [33]. In the VAERS database, there were 9,407 reports of male vaccinations from January 2006 to April 2025, with 94.7% of the individuals under 26 years of age. Among 92 positive signals, the top 3 common AEs after male vaccination were Dizziness (a = 1306, ROR = 2.60, 95%CI:2.46–2.75, P = 2.12e-258), Syncope (a = 1242, ROR = 5.92, 95%CI:5.58–6.28, P < 0.0001), and Pallor (a = 781, ROR = 6.68, 95%CI:6.21–7.20, P < 0.0001). Compared with the females, males were more likely to experience Pallor (P = 2.72e-74), Posture abnormality (P = 9.64e-29), Eye movement disorder (P = 9.64e-29), Seizure-like phenomena (P = 3.22e-22), Unresponsiveness to stimuli (P = 3.26e-20), and Immune thrombocytopenic purpura (P = 0.04) (S4 Fig).

Furthermore, 171 deaths were reported in the VAERS, among which 78.9% of the individuals received Gardasil vaccines; these individuals were under 45 years old and 97.1% were females under 30 years of age. However, on the basis of limited data, it remains impossible to establish a definite causal relationship between vaccination and death. With respect to the use of the vaccine among pregnant women, no research has been conducted to assess its impact on pregnant women. Therefore, vaccinations with this product should be avoided during pregnancy. Case reports have described premature ovarian insufficiency and infertility temporally following HPV vaccination [34, 35]. However, there are still 1,136 reports of pregnant women receiving HPV vaccination in the VAERS database, of which 99.1% received Gardasil, and 87 of the cases were severe. Among the positive PT signals, Abortion spontaneous (a = 193, ROR = 2.39, 95%CI:1.77–3.23, P = 4.91e-07), Abortion induced (a = 111, ROR = 4.79, 95%CI:2.83–8.10, P = 2.13e-08), Fetal disorder (a = 104, ROR = 3.98, 95%CI:2.41–6.57, P = 6.30e-07), and Premature labor (a = 63, ROR = 3.92, 95%CI:2.06–7.44, P = 0.0004) are particularly worthy of attention. However, such reports do not establish causality and warrant further investigation. Pregnant women should strictly follow vaccine instructions when receiving HPV vaccination.

### Global action for the elimination of cervical cancer

CCA may not show obvious symptoms in the early stages and is not easily detectable. However, in the middle or advanced stage, CCA can cause severe pain and pose a serious risk to patients’ lives. Therefore, early screening and prevention of this disease is important. The global strategic goal of the WHO to eliminate CCA is reducing the incidence rate to less than four cases per 100,000 women annually in this century. As of 2021, only 13 countries have maintained the incidence of CCA below this threshold, including 12 Middle Eastern countries in the Persian Gulf region. In 2006, the FDA approved the first HPV vaccine (Cervarix) worldwide for commercial use. Since the advent of the vaccine, several countries have successively adopted HPV vaccines in their national immunization initiatives and have gradually promoted vaccination, effectively reducing the HPV infection rate and burden of CCA.

Effective vaccination can prevent more than 90% of HPV-related cancers. At present, three HPV vaccines are available, namely, Cervarix, Gardasil, and Gardasil 9 (S2 Table). Gardasil was first approved in 2006, followed by Cervarix in 2007, whereas Gardasil 9 was introduced later in 2014 and has since become the predominant HPV vaccine in several countries, including the United States, where it has largely replaced earlier products. In contrast, Cervarix has been used in a more limited range of immunization programs and was primarily indicated for females. Differences in market availability, target age ranges, and vaccine uptake over time may therefore contribute to observed variations in reporting rates across vaccine products. Previous studies have found that HPV vaccination rates vary among countries. In 2019, the first-dose vaccination rate for women in high-income countries was 50% (95% CI: 35%–63%) and the percentage of women who completed all immunization doses was 40% (95% CI: 28%–53%). Moreover, the rate of first-dose vaccination for women in middle- and low-income countries was 16% (95% CI: 8%–31%) and the proportion of women completing all immunization doses was 12% (95% CI: 5%–24%) [33]. From a geographical perspective, only one-third of the countries in sub-Saharan Africa have added HPV vaccines to their national vaccination programs, with a first-dose vaccination rate of 33% and approximately 22% of patients completing all immunization doses. However, the incidence and death rates remain high. In Asia, the overall vaccination rate is the lowest (the first-dose vaccination rate is less than 6%, and the proportion of patients completing all vaccines is less than 4%).

### Contextual factors in vaccine uptake

While the present analysis quantified disease burden and vaccine safety signals, the population-level effectiveness of HPV vaccination is ultimately determined by coverage. Here, we review evidence from the published literature on key sociocultural, economic, and policy factors influencing vaccine uptake, which provides an essential context for addressing the implementation challenges highlighted by the present findings.

There are several limitations associated with the currently available vaccines. For example, the vaccines require strict cold-chain storage conditions, which are difficult to guarantee in areas with weak infrastructure. In addition, the vaccines are expensive, rendering them inaccessible to many people in low- and middle-income countries. The limited coverage of HPV types fails to provide protection against all high-risk HPV strains. These issues have hindered the widespread application of HPV vaccines and pose significant challenges for the prevention and control of CCA. However, the most crucial aspect is the adoption of multiple measures to increase the willingness of the population to receive the vaccine. Although adverse event reports do not represent confirmed risks, their existence and visibility may influence public confidence in vaccination programs. Therefore, understanding the characteristics of safety reporting, alongside disease burden trends, may help inform communication strategies that balance transparency with evidence-based risk interpretation.

### Policy formulation for vaccine inoculation

HPV vaccination policies vary globally. In the U.S., the Advisory Committee on Immunization Practices (ACIP) recommends HPV vaccines (approved from 2006–2015) for adolescents aged 9–26 years [36, 37], funded through school programs, private insurance, or the Vaccine for Children (VFC) CDC program for uninsured minors. In the UK. the National Health Service offers free quadrivalent HPV vaccines to 12–13-year-olds, with catch-up doses available until age 25 [38]. In 2007, Australia provided free vaccinations to all 12–13-year-old students, while Germany targeted girls aged 12–17 with free quadrivalent/bivalent vaccines. In low/middle-income countries (e.g., Malaysia and South Africa), programs focus primarily on girls aged 9–10 or school-based campaigns integrated with adolescent health initiatives [39]. China approved bivalent, quadrivalent, and nine-valent HPV vaccines between 2016 and 2018, but national immunization coverage remains absent. Limited local subsidies exist, with most costs borne by individuals.

### Implementation of the immunization program

In accordance with the monitoring of the vaccination and coverage of HPV vaccines worldwide by the WHO and UNICEF, France, Monaco, Switzerland, and the United States were the first to incorporate HPV vaccines into their national immunization programs in November 2006. As of April 2025, among the 194 member countries of the WHO, 147 countries (75.8%) have completely or partially included HPV vaccines in their national immunization programs. The proportions in the Americas (100%) and Europe (94%) are relatively high, and these regions have the best accessibility. Only 12 low-income and 34 middle-income countries have added HPV vaccines to their immunization schedules [40]. Although HPV vaccines have been licensed for more than a decade, their coverage remains unsatisfactory. For instance, in the United States, 46% of eligible adolescents failed to complete their vaccination schedule in 2019 [41].

### Reasons for vaccine hesitancy

HPV vaccination is affected by various factors such as the awareness rate, knowledge level, availability of the vaccine, affordability, coverage, quality, and degree of implementation of vaccination services.

Sociocultural Factors. 1) Expert opinion guidance. Research findings have indicated that medical experts often have a positive effect on HPV vaccination, whereas suggestions from family members, friends, and parents of other children who oppose vaccination tend to have negative effects [42]. Notably, Pediatricians and general practitioners have a higher level of understanding of the vaccine and a lower level of concern, and the recommendation rate for HPV vaccines is significantly higher [43,44]. 2) Infrastructure and health workforce. Shortages of infrastructure and health workers are an important challenge affecting the implementation of vaccination and the expansion of vaccine coverage in low- and middle-income countries because HPV vaccination is performed outside of clinical settings such as in schools, more time and resources are needed to complete the vaccination [45]. 3) Religious beliefs. Research has revealed that religious beliefs affect the willingness and behavior of patients regarding the HPV vaccination of their children [46], especially in Southeast Asia. In Indonesia, 11.3% of parents considered religion a facilitating factor in vaccination decisions [47]. In Muslim families in Malaysia, parents believe that HPV vaccination is contrary to their beliefs [48]. Some parents with religious beliefs believe that their children do not engage in premarital sex; thus, the risk of HPV infection is low, and there is no need for HPV vaccination.

Individual Factors. 1) Acquisition of knowledge regarding vaccines. The cognition and knowledge of patients about HPV vaccines are important factors affecting HPV vaccination among teenagers [42]. Fox and Rainie [49] reported that 70% of respondents stated that the information they found on the internet affected their vaccination decisions. In this information age, it is common for people to obtain first-hand knowledge from the internet. Therefore, we incorporate Google Trends analysis as a background and exploratory component to illustrate the temporal changes in public search interest related to HPV and HPV vaccination. It is worth noting that there are overlapping areas between these two trends. However, the data from Google Trends represent the standardized index of search activities and do not cover vaccination behaviors, infection rates, or the individual decision-making process. Therefore, any temporal overlap between public search interest and VAERS reports should be interpreted with caution. Such patterns may be influenced by external factors, including media attention, policy announcements, public health campaigns, or the increase in public awareness after the launch of the vaccine. However, ensuring correct scientific information is very important. 2) Perception of CCA severity. Santhanes et al. [50] reported that if a woman’s family or friends had been diagnosed with CCA, they could have a better perception of the effect of HPV infection or CCA. Compared with those who do not perceive susceptibility, they have greater willingness and demand for vaccination. 3) Consideration of vaccine safety risks. Multiple studies have reported that women with strong confidence in the prevention of cervical cancer by vaccination have increased vaccination willingness. In addition, women who doubt the efficacy of the vaccine and have concerns about its safety are less willing to vaccinate their families [51]. These findings indicate that vaccine safety is among the main obstacles to vaccination.

### Limitations

This study has limitations: First, GBD 2021 estimates may be biased because of incomplete data, particularly in low/middle-income countries with sparse original registries. Second, differences in cancer detection protocols across regions/periods may compromise cross-country comparability. Third, VAERS is a passive spontaneous reporting system and is subject to substantial underreporting, stimulated reporting, differential reporting by media attention, and variable data completeness/quality. In the present dataset, only 38,847 AE reports were recorded from 2006–2022 (24.45 per 100,000 doses) in the U.S., representing only a fraction of all postvaccination health events. Reports are not uniformly medically verified and may contain missing, inaccurate, or duplicate information; therefore, VAERS data cannot be used to estimate incidence rates or to infer causality. Fourth, important factors such as underlying comorbidities, prior medical history, concomitant medications, and coadministration of other vaccines may influence reporting but cannot be adequately measured or controlled within the VAERS. Consequently, we did not attempt causal modeling or “adjusted risk” estimation. Instead, we emphasize that the present disproportional analyses identify disproportional reporting patterns and serve the pharmacovigilance purpose of signal detection and prioritization, which should be followed by rigorous epidemiologic studies and clinical assessment. Fifth, the ecological nature of GBD data limits causal inference. While we report associations and review the literature on sociocultural factors, the present study design did not contain the necessary variables to directly test or quantify the impact of specific cultural, behavioral, or political determinants on cervical cancer outcomes. The discussion of these factors is therefore contextual and interpretive, rather than a result of the present primary analysis.

## Conclusion

This study provides a comprehensive assessment of CCA prevention from both epidemiological and post-marketing safety perspectives. Utilizing the data from the GBD 2021 study, we demonstrated that although the ASIR and ASDR of CCA worldwide decreased, the absolute number of cases and deaths remains considerable, and there are significant regional and socioeconomic disparities. These findings underscore the ongoing need for effective and equitable prevention strategies, particularly in low- and middle-income regions. On the basis of the epidemiological analysis results, the assessment of the VAERS described the reporting patterns of AEs that occurred after HPV vaccination in the actual environment. Importantly, the assessment provided descriptive insights into the reporting patterns of post-marketing safety reports, which may influence the public’s perception and acceptance of HPV vaccination programs. The GBD analysis clarified the scale and urgency of CCA prevention efforts, whereas the VAERS analysis described safety reporting patterns relevant to vaccine confidence and implementation. The combination of the two indicates that successful control of CCA requires progress in epidemiology and continuous public trust in prevention measures. Overall, our findings support the continued expansion of HPV vaccination as a cornerstone of CCA prevention, while emphasizing the need for transparent communication and evidence-based interpretation of vaccine safety data. Strengthening vaccination coverage, especially in regions with a heavy disease burden, is crucial for reducing the disparities in global CCA.

## Supporting information

S1 Table Algorithms of four disproportional analyses. (DOCX)

S2 Table The three main HPV vaccines currently available commercially. (DOCX)

S1 Fig Trend of HPV vaccination and time to onset. (TIF)

S2 Fig Safety signals for vaccine administration differ by brand. (TIF)

S3 Fig The number of new incidence and deaths cases of cervical cancer worldwide from 1990 to 2021. (TIF)

S4 Fig Volcano plot of different AEs between male and female receiving HPV vaccine injections. (TIF)
