# Supplementary material for: Global trends and forecasts of cervical cancer and a real-world safety assessment of human papillomavirus vaccines in women: A systematic analysis of the Global Burden of Disease study 2021 and the Vaccine Adverse Event Reporting System database
Source: PLoS One. 2026 Mar 23;21(3):e0345286. doi: 10.1371/journal.pone.0345286 (PMC13008108; doi:10.1371/journal.pone.0345286)
Supplement: S1 Table — (DOCX) [file pone.0345286.s001.docx]

**S1 Table. Algorithms of four disproportional analyses.**

| Algorithms | | Equation | Criteria |
| --- | --- | --- | --- |
| ROR | $\text{ ROR=}\frac{\text{a / c}}{b/d}$ $ROR 95\%CI=e^{\ln(ROR)\pm1.96\sqrt{(\frac{1}{a}+\frac{1}{b}+\frac{1}{c}+\frac{1}{d})}}$   | | $a\geq3$ $lower \lim it of 95\%CI>1$   |
| PRR | $PRR=\frac{a/(a+b)}{c/(c+d)}$ $\chi^{2}=(ad-bc)^{2}(a+b+c+d)/[(a+b)(c+d)(a+c)(b+d)]$ $PRR 95\%CI=e^{\ln(PRR)\pm1.96\sqrt{(\frac{1}{a}-\frac{1}{a+b}+\frac{1}{c}-\frac{1}{c+d})}}$     | | $\text{a}\geq3$ $\text{PRR}\geq2$ $\chi^{2}\geq4$     |
| BCPN | $\alpha=\alpha_{1}+\alpha_{2}=2$ $\beta=\beta_{1}+\beta_{2}=2$ $N=a+b+c+d$ $\gamma=\gamma_{11}\frac{(N+\alpha)(N+\beta)}{(a+b+\alpha_{1})(a+c+\beta_{1})}$ $IC=\log_{2}\frac{a(a+b+c+d)}{(a+b)(a+c)}$ $E(IC)=\log_{2}\frac{(a+\gamma_{11})(N+\alpha)(N+\beta)}{(N+\gamma)(a+b+\alpha_{1})(a+c+\beta_{1})}$ $V(IC)\approx\left(\frac{1}{\ln2}\right)^{2}\left[\begin{matrix} \frac{N-a+\gamma-\gamma_{11}}{(a+\gamma_{11})(1+N+\gamma)} \\ +\frac{N-a-b+\alpha-\alpha_{1}}{(a+b+\alpha_{1})(1+N+\alpha)} \\ +\frac{N-a-c+\beta-\beta_{1}}{(a+c+\beta_{1})(1+N+\beta)} \end{matrix}\right]$ $\alpha_{1}=\beta_{1}=1;\gamma_{11}=1$               | | $a\geq3$ $IC-2SD>0$ $SD=V(IC)^{0.5}$     |
| MGPS | $EBGM=a(a+b+c+d)/[(a+c)(b+d)]$ $EBGM05=e^{\ln(EBGM)\pm1.96\sqrt{\frac{1}{a}+\frac{1}{b}+\frac{1}{c}+\frac{1}{d}}}$   | | $a>0$ $EBGM05>2$   |

Abbreviations: EBGM, empirical Bayesian geometric mean; IC, information component; SD, standard deviation; IC-2SD, lower limit of the 95% CI of the IC; E (IC), IC expectations; V (IC), variance of IC; EBGM05, lower limit of the 95% CI of the EBGM; 95% CI, 95% confidence interval.
