# Supplementary material for: Global trends and forecasts of cervical cancer and a real-world safety assessment of human papillomavirus vaccines in women: A systematic analysis of the Global Burden of Disease study 2021 and the Vaccine Adverse Event Reporting System database
Source: PLoS One. 2026 Mar 23;21(3):e0345286. doi: 10.1371/journal.pone.0345286 (PMC13008108; doi:10.1371/journal.pone.0345286)
Supplement: S2 Table — (DOCX) [file pone.0345286.s002.docx]

**S2 Table. The three main HPV vaccines currently available commercially.**

| Brand name | Cervarix® | Gardasil®4 | Gardasil®9 |
| --- | --- | --- | --- |
| Type | Divalent | Quadrivalent | Nine-valent |
| Manufacturers | GSK Plc | Merck Sharp & Dohme | Merck Sharp & Dohme |
| Global time to Market | 2009 | 2006 | 2014 |
| Target population | Female  aged 9-25 years | Male/Female  aged 9-26 years | Male/Female  aged 9-45 years |
| Corresponding to HPV virus type |  |  |  |
| High-risk HPV | 16/18 | 16/18 | 16/18/31/33/45/52/58 |
| Low-risk HPV |  | 6/11 | 6/11 |
| Dose of inoculation | 3 doses  or 2 doses in 9-14 years | 3 doses | 3 doses  or 2 doses in 9-14 years |
| Interval between inoculation | On the first day, one month later, six months later | On the first day, two months later, six months later | On the first day, two months later, six months later |
